# Impact of Serum Lactate as an Early Serum Biomarker for Cardiopulmonary Parameters within the First 24 Hours of Intensive Care Unit Treatment in Patients with Isolated Traumatic Brain Injury

**DOI:** 10.3390/diagnostics13101777

**Published:** 2023-05-17

**Authors:** Michael Bender, Michaela Friedrich, Hans Voigtmann, Kristin Haferkorn, Eberhard Uhl, Marco Stein

**Affiliations:** 1 Department of Neurosurgery, Justus-Liebig-University, 35392 Gießen, Germany; 2 Department of Neurosurgery, Hospital Aschaffenburg-Alzenau, 63739 Aschaffenburg, Germany

**Keywords:** serum lactate, traumatic brain injury, cardiopulmonary parameters, intensive care unit treatment

## Abstract

Objective: Cardiopulmonary (CP) complications are well-known phenomena in patients with isolated traumatic brain injury (iTBI) that can lead to tissue hypoperfusion and hypoxia. Serum lactate level is a well-known biomarker, indicating these systemic dysregulations in various diseases, but this has not been investigated in iTBI patients so far. The current study evaluates the association between serum lactate levels upon admission and CP parameters within the first 24 h of intensive care unit (ICU) treatment in iTBI patients. Patients and Methods: 182 patients with iTBI who were admitted to our neurosurgical ICU between December 2014 and December 2016 were retrospectively evaluated. Serum lactate levels on admission, demographic, medical, and radiological data upon admission, as well as several CP parameters within the first 24 h of ICU treatment, were analyzed, as well as the functional outcome at discharge. The total study population was dichotomized into patients with an elevated serum lactate level (lactate-positive) and patients with a low serum lactate level (lactate-negative) upon admission. Results: 69 patients (37.9%) had an elevated serum lactate level upon admission, which was significantly associated with a lower Glasgow Coma Scale score (*p* = 0.04), a higher head AIS score (*p* = 0.03), and a higher Acute Physiology and Chronic Health Evaluation II score (*p* = 0.01) upon admission, as well as a higher modified Rankin Scale score (*p* = 0.002) and a lower Glasgow Outcome Scale score (*p* < 0.0001) at discharge. Furthermore, the lactate-positive group required a significantly higher norepinephrine application rate (NAR; *p* = 0.04) and a higher fraction of inspired oxygen (FiO2; *p* = 0.04) to maintain the defined CP parameters within the first 24 h. Conclusion: ICU-admitted iTBI patients with elevated serum lactate levels upon admission required higher CP support within the first 24 h of ICU treatment after iTBI. Serum lactate may be a helpful biomarker for improving ICU treatment in the early stages.

## 1. Introduction

One of the most important aspects of intensive care unit (ICU) treatment for patients with isolated traumatic brain injury (iTBI) within the first 24 h is to avoid intracranial hypertension, as well as systemic hypotension, hypoxia, and reduced cerebral perfusion, which is frequently caused by the initial injury to the brain [1,2]. However, additional extra-cerebral organ dysfunctions (e.g., acute kidney injury, sepsis, pulmonary edema, or cardiac complications) can lead to reduced cerebral perfusion and oxygenation, causing additional cerebral injury in this vulnerable time period [2,3,4,5,6,7]. Well-known phenomena in iTBI patients are electrocardiographic changes and cardiac wall motion abnormalities, as well as transient left ventricular dysfunction, which is named neurogenic stunned myocardium [7,8,9]. Nevertheless, the pathophysiological pathway of a neurogenic stunned myocardium remains unclear. However, it may be induced by an excessive systematic release of catecholamines into the circulation and an acute inflammatory response, which could lead to systemic and pulmonary vasoconstriction, reduced cardiac output, and pulmonary capillary leakage [2,7,8,9,10,11,12,13,14,15]. These cardiopulmonary (CP) complications can lead to hypotension, tissue hypoperfusion, hypoxia, and anaerobic metabolic conditions, which may be expressed by an increased serum lactate level.

In general, an increased serum lactate level is frequently a consequence of increased production (e.g., anaerobic metabolic conditions due to hypoxia or tissue hypoperfusion) and/or decreased elimination (e.g., liver dysfunction) [16]. Moreover, an increased serum lactate level could be the result of several drugs, intoxication, seizure, renal failure, mesenteric ischemia, or high-dose catecholamine therapy. The serum lactate level upon admission is a well-known and commonly used biomarker to evaluate disease severity and predict outcome in patients suffering from cardiac arrest, sepsis, septic shock, liver dysfunction, regional ischemia, or general trauma, as well as traumatic brain injury [16,17,18,19,20,21,22,23,24,25]. Therefore, serum lactate levels upon admission may be a helpful biomarker for early identification of patients with a potentially increased requirement of pulmonary and circulation support within the first 24 h. However, the impact of increased serum lactate levels upon admission on CP parameters within the first 24 h of ICU-admitted iTBI patients is still unknown. Therefore, the present study aimed to evaluate the impact of serum lactate as an early serum biomarker for CP parameters within the first 24 h of ICU treatment in iTBI patients.

## 2. Materials and Methods

### 2.1. Study Design and Population

All patients with iTBI who were treated for at least 24 h at our neurosurgical ICU from December 2014 to December 2016 (*n* = 182) were analyzed retrospectively. The presence of iTBI was confirmed on admission by a computed tomography (CT) scan in the emergency department. Patients with acute cardiac and pulmonary decompensation, aged under 18 years, and with an extracranial injury, defined as an Abbreviated Injury Scale (AIS), score of ≥2 to any other body region were excluded [26]. The study protocol was approved by the ethical committee of Justus-Liebig-University (No: 93/20). The baseline data, CP parameters, serum biomarkers, treatment regimes, radiological data, and clinical outcome at discharge were extracted from the medical records and analyzed.

### 2.2. General Intensive Care Unit Management

All patients were primarily admitted to the emergency department at our university hospital. After clinical examination and verification of the iTBI diagnosis on the CT scan, the patients were transferred immediately, or after urgent surgical treatment, to our neurosurgical ICU for at least 24 h. The CP monitoring routinely included an invasive blood pressure measurement catheter (Combitrans Monitoring Set; B. Braun, Melsungen, Germany), a pulse oximeter (Nellcor adult SpO_2_ sensor; Covidien LLC, Mansfield, MA, USA), and a 3-lead electrocardiogram (B. Braun, Melsungen, Germany). Additionally, all patients received a central venous catheter (Arrow International, Inc., Reading, PA, USA). Arterial blood samples were drawn at four-hour intervals for blood gas analysis (ABL800 FLEX; Radiometer, Copenhagen, Denmark and Krefeld, Germany). The CP parameter targets were defined as systolic arterial blood pressure between 120 mmHg and 140 mmHg, and arterial oxygen partial pressure (PaO_2_) ≥ 100 mmHg, independent of a surgical or conservative procedure. In terms of respiratory insufficiency, or a Glasgow Coma Scale (GCS), score of less than 9, endotracheal intubation and mechanical ventilation (Servo-I; Maquet, Rastatt, Germany) were indicated. All intubated and mechanically ventilated patients were sedated with sufentanil (35–100 µg/h) and midazolam (5–40 mg/h) or propofol (200–500 mg/h) to a Richmond Agitation-Sedation Scale (RAAS) score of −4 within the first 24 h of ICU treatment after the iTBI [27]. Additionally, intracranial pressure (ICP) was measured in these patients with an intraparenchymal pressure probe (Codman Microsensor Basic Kit, Raynham, MA, USA) until the patients were neurologically assessable. An ICP value < 20 mmHg was adjusted in all iTBI patients.

### 2.3. Baseline Data

The baseline data included demographic data, the body mass index (BMI), the GCS, the head AIS, and Acute Physiology and Chronic Health Evaluation II (APACHE II) scores upon admission [26,28,29]. The patients were stratified into mild iTBI (GCS = 13–15), moderate iTBI (GCS = 9–12), and severe iTBI (GCS = 3–8) groups. Comorbidities were analyzed regarding preexisting chronic arterial hypertension, diabetes mellitus, chronic pulmonary disease, chronic renal insufficiency, chronic heart failure, and chronic cardiac arrythmia. Additionally, evaluation of premedication included long-term medication with beta-blockers, antihypertensive, antiobstructive, and antidiabetic drugs, antiplatelet agents, new oral anticoagulants, and vitamin K antagonists.

### 2.4. Cardiopulmonary Parameters

The average fraction of inspired oxygen (FiO_2_), the average norepinephrine application rate (NAR), mean arterial blood pressure, heart rate, intubation status, PaO_2_, positive end expiratory pressure (PEEP) within the first 24 h of ICU treatment, and body temperature, as well as the presence of aspiration, were assessed as CP parameters. Aspiration was defined as a radiological diagnosis from the chest *x*-ray, or as a clinical diagnosis, due to endotracheal suction of the patients. The CP parameters were continuously recorded at five-minute intervals and stored in the digital ICU data recording system.

### 2.5. Serum Biomarkers

Blood samples were immediately drawn upon admission. The serum lactate levels in mmol/L (ADVIA Chemistry XPT^®^ LAC Assay, Siemens, Germany; reference level 0.5.2.2 mmol/L) were assessed as a serum biomarker for CP parameters. Furthermore, C-reactive protein (CRP) in mg/L (ADVIA Chemistry XPT^®^, Siemens, Germany), white blood cell count in giga/L (XE 5000 Hematology Analyzer, Sysmex, Germany), hemoglobin levels in g/dL (XE 5000 Hematology Analyzer, Sysmex, Germany), hematocrit levels in L/L (XE 5000 Hematology Analyzer, Sysmex, Germany), cholinesterase in U/L (ADVIA Chemistry XPT^®^, Siemens, Germany), blood glucose levels in mg/dL (ADVIA Chemistry XPT^®^, Siemens, Germany), pH values (ABL800 FLEX; Radiometer, Copenhagen, Denmark and Krefeld, Germany), albumin levels in g/L (ADVIA Chemistry XPT^®^, Siemens, Germany), creatinine in mg/dL (ADVIA Chemistry XPT Crea assay, Siemens, Germany), cortisol levels in µg/dL (ADVIA Centaur XPT^®^, Siemens, Germany), and troponin I in µg/dL (ADVIA Centaur XPT^®^, Siemens, Germany) upon admission were analyzed in the entire study population.

### 2.6. Treatment Regime

The treatment of all iTBI patients was categorized into medical and surgical treatment procedures. Surgical treatments consisted of insertion of an intraparenchymal pressure probe, insertion of an external ventricular drain (Neuromedex GmbH, Hombrechtikon, Switzerland), evacuation of an epidural, subdural, or intracerebral hematoma or contusion hemorrhage, and/or a decompressive craniectomy. In contrast, medical treatments comprised all the procedures of conservative ICU treatment.

### 2.7. Radiological Data

The initial CT scan upon admission was assessed by two independent neurosurgical consultants (M.B. and M.F.) and stratified with respect to the iTBI entity: subdural hematoma, epidural hematoma, traumatic subarachnoid hemorrhage, contusion hemorrhage, diffuse axonal injury, isolated skull fracture, and diffuse/combined iTBI (≥2 entities of TBI, as mentioned above).

### 2.8. Clinical Outcome at Discharge

The modified Rankin Scale (mRS) and Glasgow Outcome Scale (GOS) scores were used to assess the clinical outcome at discharge [30,31]. Additionally, the length of the ICU and overall hospital stay was analyzed.

### 2.9. Statistical Analysis

The primary endpoint of the study was to evaluate the impact of serum lactate as an early serum biomarker for CP parameters within the first 24 h of ICU treatment in iTBI patients. Therefore, the entire study population was dichotomized into patients with a serum lactate level > 1.86 mmol/L (lactate-positive) and patients with a serum lactate level ≤ 1.86 mmol/L (lactate-negative). The cutoff level was calculated using the average serum lactate level of the entire study population. The data are expressed as the median and interquartile range (IQR) for the parameters with non-normal distributions, and as the mean ± standard deviation in case of normal distribution. The chi-squared test and *t*-test were performed to compare the groups. The Statistical Package for the Social Sciences for Windows (Version 15.0; SPSS Inc., Chicago, IL, USA) and Prism Version 5 statistical software (GraphPad Software, Inc., La Jolla, CA, USA) were used for the data analysis. A *p*-value < 0.05 was defined as the level of significance.

## 3. Results

### 3.1. Main Characteristics

In total, 182 patients (69 women and 113 men) with a mean age of 65.9 ± 20.1 years could be included. A median GCS score of 11 (IQR 3–14), a median head AIS score of 1 (IQR1–5), and a median APACHE II score of 15 (IQR 9–22) were recorded upon admission. The most common comorbidity was the presence of chronic arterial hypertension, and the most common premedication was the intake of antihypertensive drugs. A total of 93 patients (51.1%) were intubated and mechanically ventilated. A mean FiO_2_ of 31.7 ± 13 and a NAR of 0.06 ± 0.27 µg/kg/min were required to reach the CP targets within the first 24 h of ICU treatment. The median arterial blood pressure was 82 mmHg (IQR 74–90), the median heart rate was 75 beats per minute (IQR 67–86), the median PEEP level was 5 (IQR 5–7), and the median PaO_2_ was 155 mmHg (IQR 106–229) within the first 24 h of ICU treatment. Additionally, the median body temperature upon admission was 36.6 °C (IQR 36.2–37.2), and 53 patients (29.1%) had aspirated, in accordance with our definition. Medical treatment was performed in 95 patients (52.2%), and 47.8% of the iTBI patients were treated surgically. A diffuse/combined iTBI was the most frequent injury (68.7%) seen on CT. The median length of the overall hospital stay was 12 days (IQR 5–24), and 7 days (IQR 2–22) for ICU treatment. The intra-hospital mortality rate was 30.2%, the median mRS at discharge was 3 (IQR 0–6), and the median GOS was 4 (IQR 1–5), as shown in Table 1.

### 3.2. Serum Lactate

**Figure 1:**
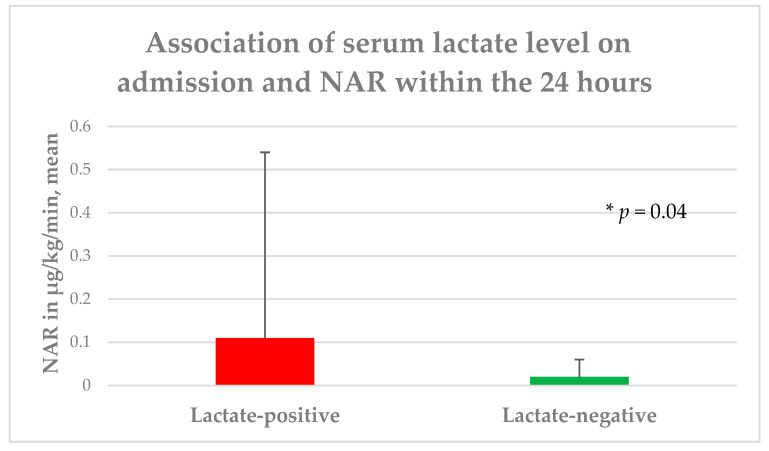
Association of serum lactate level on admission and NAR within the first 24 h; NAR: norepinephrine application rate.

**Figure 2:**
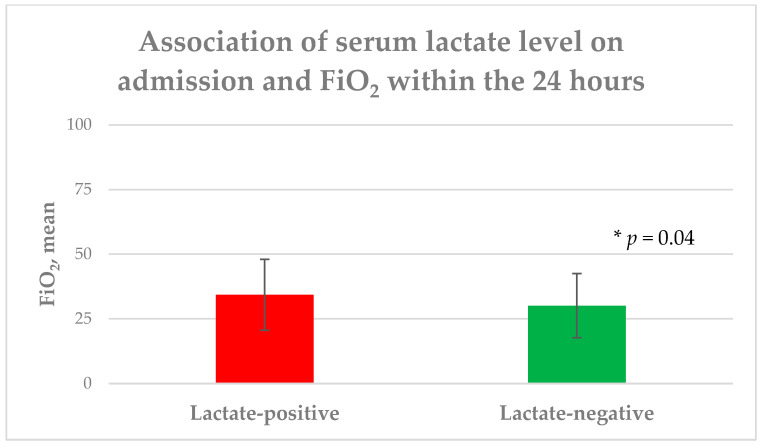
Association of serum lactate level on admission and FiO_2_ within the first 24 h; FiO_2_: inspiratory oxygen fraction.

An initially elevated serum lactate level was measured in 69 patients (37.9%), while 113 patients (62.1%) showed no increased serum lactate levels. In the univariate analysis, no significant difference between the groups was found in regard to age (*p* = 0.29), gender (*p* = 0.5), BMI (*p* = 0.77), or the presence of severe iTBI (*p* = 0.35) on admission. Additionally, the groups did not differ statistically significantly, with respect to comorbidities and premedication, treatment regimen, radiological data, and length of hospital stay. The lactate-positive group had a significantly lower pH level (7.39 ± 0.1) upon admission in contrast to the lactate-negative group (7.41 ± 0.1), albeit without a circulation-relevant acidosis < 7.35 in both groups (*p* = 0.02). Moreover, the patients with initially raised serum lactate levels had a significantly lower GCS (*p* = 0.04) and rate of mild iTBI (*p* = 0.0005), as well as a higher head AIS (*p* = 0.03), a higher APACHE II score (*p* = 0.01), and a higher rate of moderate iTBI (*p* = 0.02). With respect to the primary endpoint of the study, the patients with initially elevated serum lactate levels required a higher NAR (*p* = 0.04) and FiO_2_ (*p* = 0.04) to achieve the CP targets within the first 24 h of ICU treatment (Figure 1 and Figure 2). No significant differences between the groups, concerning mean arterial blood pressure (*p* = 0.13), rate of patients requiring NAR (*p* = 0.15), heart rate (*p* = 0.78), number of intubated patients (*p* = 0.25), PEEP level (*p* = 0.86), and PaO_2_ (*p* = 0.31) within the first 24 h, or presence of aspiration (*p* = 0.1) and body temperature (*p* = 0.07) upon admission were found. In contrast, the patients with an initially raised serum lactate level had a significantly higher mortality rate (*p* = 0.0007), as well as higher mRS (*p* = 0.002) and lower GOS (*p* < 0.0001) at discharge (Table 1).

## 4. Discussion

### 4.1. Summary of Findings

This retrospective study was conducted on 182 patients to evaluate the impact of serum lactate levels on CP parameters among ICU-admitted patients with iTBI. The patients with initially elevated serum lactate levels upon admission had a significantly lower GCS, a lower rate of mild iTBI, and lower pH levels, as well as higher head AIS scores, higher APACHE II scores, and a higher rate of moderate iTBI upon admission. These iTBI patients also exhibited a significantly higher mortality rate, as well as a higher mRS, and a lower GOS at discharge. In addition, the patients with initially raised serum lactate levels required a significantly higher NAR and FiO2 to reach the CP target within the first 24 h of ICU treatment. The serum lactate levels upon admission may be a helpful serum biomarker to optimize ICU treatment, particularly concerning the early identification of patients who could potentially develop CP complications within the first 24 h of ICU treatment.

### 4.2. Serum Lactate Levels

The lactate level is an important parameter in human metabolism that is produced in almost all human tissues, frequently in the human muscle, and eliminated primarily by the liver and, to a lesser amount, by the kidneys [5]. Under aerobic conditions, glycolysis results in pyruvate, which subsequently passes the Krebs cycle without a relevant production of lactate. However, lactate is the end product of glycolysis in anaerobic metabolism, followed by entering the Cori cycle as a metabolite for gluconeogenesis [32,33,34]. The serum lactate level is an easy-to-determine biomarker from blood samples that is routinely used in ICU treatment to evaluate the severity of the underlying diseases, as well as the occurrence of ischemia, hypoxia, and tissue hypoperfusion in a variety of diseases [16,17,18,19,20,21,22]. The cutoff level for serum lactate in the present study was 1.86 mmol/L, which is comparable to those of previous studies [2,3]. The iTBI patients with elevated serum lactate levels upon admission had a lower rate of mild iTBI, a higher rate of moderate iTBI upon admission, and higher intra-hospital mortality, as well as a higher mRS and lower GOS at discharge, which is not surprising, because these patients had a significantly lower GCS, a higher head AIS score, and a higher APACHE II score upon admission. In addition, the patients with initially raised serum lactate levels had a higher rate of severe iTBI (48.8% vs. 40.7%). However, this difference did not reach the level of significance (*p* = 0.35), which could be explained by the study’s limited sample size. The lactate-positive group exhibited a significantly lower pH level than the lactate-negative group (7.39 ± 0.1 vs. 7.41 ± 0.1), however without a relevant effect on the systemic circulation, due to an acidosis-induced NAR resistance. Moreover, no statistically significant differences according to comorbidities and premedication, treatment regime, radiological data, or length of hospital stay were found between the groups.

### 4.3. Cardiopulmonary Parameters

All included iTBI patients were treated in the ICU for at least 24 h without any significant difference with respect to arterial blood pressure, the depth of sedation of intubated patients, the rate of patients requiring norepinephrine, heart rate, the number of intubated patients, PEEP level, and PaO_2_ within the first 24 h, as well as the presence of aspiration and body temperature upon admission. However, the patients with elevated serum lactate levels upon admission required a significantly higher NAR and FiO_2_ to reach the desired systolic pressure and arterial oxygen pressure targets. Apart from the lower GCS score, as well as the higher head AIS and APACHE II scores in the group of patients with initial elevated serum lactate levels, the systolic blood pressure, PaO_2_, and depth of sedation targets in the intubated patients (RAAS = −4) were equal in all the ICU-admitted iTBI patients within the first 24 h, so that, in our mind, both groups were assimilable, concerning the NAR and FiO_2_.

These findings suggest that an elevated serum lactate level upon admission could be a useful serum biomarker for the risk assessment of iTBI patients with a potentially higher need for CP support within the first 24 h of ICU treatment. Therefore, those patients may benefit from early extended hemodynamic and pulmonary monitoring, for example, by using a Swan-Ganz catheter and/or pulse contour cardiac output system (PiCCO) and/or intermittent or continuous transthoracic/transesophageal echocardiography [35]. Nevertheless, serum lactate is a cardiopulmonary parameter by itself and should always interpreted in conjunction with other parameters. With this background in mind, no general cause effect can be extrapolated.

### 4.4. Limitations and Strengths of the Study

The present study has several limitations, so the results should be interpreted with caution. First, the single-center and retrospective character of the study has well-known limitations. Second, only a limited number of CP complications within the first 24 h of ICU treatment were assessed. The assessment of additional pulmonary and cardiac parameters (e.g., global end-diastolic volume index, cardiac index, pulmonary vascular permeability index, and extravascular lung water) could be executed by using an extended CP monitoring system, such as the PiCCO system [35]. However, the most common indication for the implantation of a PiCCO system in our ICU is the presence of sepsis and/or septic shock. Furthermore, this is purely a descriptive study with a small sample size, so that no causal effect can be inferred from the results of the current study. Moreover, due to the retrospective character of the study, it was not possible to analyze the exact time period from admission to lactate collection. Finally, 3-lead electrocardiography was applied and continuously monitored in all iTBI patients; however, it was not digitally monitored. Therefore, retrospective analysis was not practicable.

In contrast, the strength of the present study is the data set with complete demographic, clinical, CP, laboratory chemistry, and radiological data. Moreover, to the best of our knowledge, this is the first report evaluating the association of serum lactate levels with CP parameters within the first 24 h of ICU treatment in iTBI patients.

Regardless of the study’s limitations, the findings may help improve ICU treatment in iTBI patients, especially concerning the early identification of patients with a potentially higher need for CP support within the first 24 h. Patients with initially elevated serum lactate levels upon admission may potentially benefit from extended hemodynamic and pulmonary monitoring.

## 5. Conclusions

The present study investigated the impact of elevated serum lactate levels upon admission on CP parameters within the first 24 h of ICU treatment in iTBI patients. An elevated serum lactate level was associated with a lower GCS, a lower rate of mild iTBI, a higher head AIS score, a higher APACHE II score, and a higher rate of moderate iTBI upon admission. Moreover, the iTBI patients with initially raised serum lactate levels required a significantly higher NAR and FiO_2_ to reach the CP targets within the first 24 h. Therefore, serum lactate could be a helpful serum biomarker for improving ICU treatment, especially regarding the early identification of iTBI patients with a higher need for pulmonary and circulation support.

## Figures and Tables

**Table 1:** Baseline data of the study population and univariate analysis of the serum lactate level.

Parameters	All Patients (*n* = 182)	Lactate-Positive (*n* = 69)	Lactate-Negative (*n* = 113)	*p*-Value
Baseline data				
Age, years, mean (SD) *	65.9 (20.1)	63.9 (20.4)	67.1 (19.9)	0.29
Women, *n* (%) *	69 (35.6)	24 (34.8)	45 (39.8)	0.5
Men, *n* (%) *	113 (58.2)	45 (65.2)	68 (60.2)	
BMI, kg/m^2^, median (IQR) *	25 (23.4–27.8)	24.8 (23.1–28)	25.1 (23.7–27.8)	0.77
GCS score, median (IQR) *	11 (3–14)	10 (3–13)	12 (3–15)	0.04
Mild iTBI (GCS = 13–15), *n* (%) *	71 (39)	18 (26.1)	53 (46.9)	0.005
Moderate iTBI (GCS = 9–12), *n* (%) *	32 (17.6)	18 (26.1)	14 (12.4)	0.02
Severe iTBI (GCS = 3–8), *n* (%) *	79 (43.4)	33 (47.8)	46 (40.7)	0.35
Head AIS, median (IQR) *	1 (1–5)	5 (3–5)	2 (1–5)	0.03
APACHE II, median (IQR) *	15 (9–22)	18 (11–24)	13 (9–20.5)	0.01
Comorbidities and premedication				
Chronic arterial hypertension, *n* (%) *	93 (51.1)	32 (46.4)	61 (54)	0.32
Diabetes mellitus, *n* (%) *	25 (13.7)	7 (10.1)	18 (15.9)	0.27
Chronic pulmonary disease, *n* (%) *	19 (10.4)	8 (11.6)	11 (9.7)	0.69
Chronic renal insufficiency, *n* (%) *	15 (8.2)	4 (5.8)	11 (9.7)	0.35
Chronic heart failure, *n* (%) *	10 (5.5)	5 (7.2)	5 (4.4)	0.42
Chronic cardiac arrythmia, *n* (%) *	35 (19.2)	12 (17.4)	23 (20.4)	0.62
Beta-blockers, *n* (%) *	61 (33.5)	19 (27.5)	42 (37.2)	0.18
Antihypertensive drugs, *n* (%) *	72 (39.6)	23 (33.3)	49 (43.4)	0.18
Antiobstructive drugs, *n* (%) *	17 (9.3)	7 (10.1)	10 (8.9)	0.77
Antidiabetic drugs, *n* (%) *	16 (8.8)	4 (5.8)	12 (10.6)	0.27
Antiplatelet agents, *n* (%) *	38 (20.9)	10 (14.5)	28 (24.8)	0.13
New oral anticoagulants, *n* (%) *	12 (6.6)	6 (8.7)	6 (5.3)	0.37
Vitamin K antagonist, *n* (%) *	26 (14.3)	8 (11.6)	18 (15.9)	0.42
Cardiopulmonary parameters				
NAR, µg/kg/min, mean (±SD) **	0.06 (0.27)	0.11 (0.43)	0.02 (0.04)	0.04
Requiring norepinephrine **				
Yes, *n* (%)	80 (44)	35 (50.7)	45 (39.8)	0.15
No, *n* (%)	102 (56)	34 (49.3)	68 (60.2)	
Mean arterial blood pressure, mmHg, median (IQR) **	82 (74–90)	80 (72.3–89.5)	83 (74–90)	0.13
Heart rate, beats per minute, median (IQR) **	75 (67–86)	75 (68–87)	76 (65.3–86)	0.76
FiO_2_, mean (±SD) **	31.7 (13)	34.3 (13.7)	30.1 (12.4)	0.04
Intubated patients, *n* (%) **	93 (51.1)	39	54	0.25
PEEP level, median (IQR) **	5 (5–7)	5 (5–7)	5 (5–7)	0.86
PaO_2_, mmHg, median (IQR) **	155 (106–229)	182 (120–243.8)	143 (99–229)	0.31
Aspiration, *n* (%) *	53 (29.1)	25 (36.2)	28 (24.8)	0.1
Body temperature, °C, median (IQR) *	36.6 (36.2–37.2)	36.5 (37.7–37.2)	36.7 (36.2–37.3)	0.07
Biomarkers				
CRP, mg/L, mean (±SD) *	20.2 (37.9)	11.3 (25.1)	25.7 (43.2)	0.1
White blood cells, Gg/L, mean (±SD) *	12.1 (6.5)	13 (6.7)	11.7 (6.4)	0.17
Hemoglobin, g/dL, mean (SD) *	13 (2.3)	12.8 (2.6)	12.8 (2.1)	0.95
Hematocrit, %, mean (SD) *	37.5 (6.2)	37.6 (6.6)	37.4 (5.9)	0.89
Cholinesterase, U/L, mean (±SD) *	7404.7 (2748.9)	7815.3 (2791.7)	7151.8 (2703.8)	0.12
Blood glucose, mg/dL, mean (±SD) *	141.7 (41.7)	149.2 (48.2)	137.2 (36.8)	0.06
pH level, mean (±SD) *	7.4 (0.1)	7.39 (0.1)	7.41 (0.1)	0.02
Albumin, g/L, mean (SD) *	38.3 (6.6)	38.1 (7.8)	38.3 (5.7)	0.84
Creatinine, mg/dL, mean (SD) *	1 (0.8)	1.1 (0.9)	0.9 (0.6)	0.22
Cortisol, µg/dL, mean (±SD) *	29.3 (19.8)	30.8 (20)	28.5 (19.8)	0.52
Troponin I, µg/dL, mean (±SD) *	0.2 (1.9)	0.09 (0.21)	0.37 (2.5)	0.38
Treatment regime				
Medical treatment, *n* (%)	95 (52.2)	30 (43.5)	65 (57.5)	0.07
Surgical treatment, *n* (%)	87 (47.8)	39 (56.5)	48 (42.5)	
Radiological data				
Subdural hematoma, *n* (%) *	36 (19.8)	12 (17.4)	24 (21.2)	0.53
Epidural hematoma, *n* (%) *	4 (2.2)	1 (1.4)	3 (2.7)	0.59
Traumatic SAH, *n* (%) *	10 (5.5)	4 (5.8)	6 (5.3)	0.89
Contusion hemorrhage, *n* (%) *	4 (2.2)	0 (0)	4 (3.5)	0.11
Diffuse axonal injury, *n* (%) *	2 (1.1)	0 (0)	2 (1.8)	0.27
Isolated skull fracture, *n* (%) *	1 (0.6)	0 (0)	1 (0.9)	0.43
Diffuse/combined iTBI, *n* (%) *	125 (68.7)	52 (75.4)	73 (64.6)	0.13
Intra-hospital outcome				
Hospital stay, overall days, median (IQR) ***	12 (5–24)	14 (5.5–24)	12 (5–24.5)	0.74
Hospital stay, ICU days, median (IQR) ***	7 (2–22)	8 (2–23)	6 (2–20.5)	0.97
Survivors, *n* (%) ***	127 (69.8)	38 (55.1)	89 (78.8)	0.0007
Non-survivors, *n* (%) ***	55 (30.2)	31 (44.9)	24 (21.2)	
mRS, median (IQR) ***	3 (0–6)	5 (0–6)	3 (0–5)	0.002
GOS, median (IQR) ***	4 (1–5)	2 (1–5)	4 (2–5)	<0.0001

SD: standard deviation, BMI: body mass index, IQR: interquartile range, GCS: Glasgow Coma Scale, iTBI: isolated traumatic brain injury, AIS: abbreviated injury scale, APACHE II: Acute Physiology and Chronic Health Evaluation II, NAR: norepinephrine application rate, FiO2: fraction of inspired oxygen, PEEP: positive end expiratory pressure, CRP: C-reactive protein, SAH: subarachnoid hemorrhage, ICU: intensive care unit, mRS: modified Rankin scale, GOS: Glasgow Outcome Scale. * On admission, ** Within the first 24 h, *** At discharge.

## Data Availability

The data presented in this study are available on request from the corresponding author.
